# The Potential Role of Sensors, Wearables and Telehealth in the Remote Management of Diabetes-Related Foot Disease

**DOI:** 10.3390/s20164527

**Published:** 2020-08-13

**Authors:** Jonathan Golledge, Malindu Fernando, Peter Lazzarini, Bijan Najafi, David G. Armstrong

**Affiliations:** 1 Ulcer and wound Healing consortium (UHEAL), Queensland Research Centre for Peripheral Vascular Disease, College of Medicine and Dentistry, James Cook University, Townsville, Queensland 4811, Australia; malindu.fernando@my.jcu.edu.au; 2 The Department of Vascular and Endovascular Surgery, Townsville University Hospital, Townsville, Queensland 4814, Australia; 3 School of Public Health and Social Work, Queensland University of Technology, Brisbane, Queensland 4000, Australia; Peter.Lazzarini@health.qld.gov.au; 4 Allied Health Research Collaborative, Metro North Hospital and Health Service, Brisbane, Queensland 4006, Australia; 5 Interdisciplinary Consortium on Advanced Motion Performance (iCAMP), Michael E. DeBakey Department of Surgery, Baylor College of Medicine, Houston, TX 77030, USA; bijan.najafi@bcm.edu; 6 Southwestern Academic Limb Salvage Alliance (SALSA), Department of Surgery, Keck School of Medicine of University of Southern California, Los Angeles, CA 90089, USA; armstrong@usa.net

**Keywords:** diabetic foot, remote-monitoring, sensors, prevention, telehealth, peripheral artery disease, diabetic peripheral neuropathy, remote patient monitoring

## Abstract

Diabetes-related foot disease (DFD), which includes foot ulcers, infection and gangrene, is a leading cause of the global disability burden. About half of people who develop DFD experience a recurrence within one year. Long-term medical management to reduce the risk of recurrence is therefore important to reduce the global DFD burden. This review describes research assessing the value of sensors, wearables and telehealth in preventing DFD. Sensors and wearables have been developed to monitor foot temperature, plantar pressures, glucose, blood pressure and lipids. The monitoring of these risk factors along with telehealth consultations has promise as a method for remotely managing people who are at risk of DFD. This approach can potentially avoid or reduce the need for face-to-face consultations. Home foot temperature monitoring, continuous glucose monitoring and telehealth consultations are the approaches for which the most highly developed and user-friendly technology has been developed. A number of clinical studies in people at risk of DFD have demonstrated benefits when using one of these remote monitoring methods. Further development and evidence are needed for some of the other approaches, such as home plantar pressure and footwear adherence monitoring. As yet, no composite remote management program incorporating remote monitoring and the management of all the key risk factors for DFD has been developed and implemented. Further research assessing the feasibility and value of combining these remote monitoring approaches as a holistic way of preventing DFD is needed.

## 1. Introduction

Diabetes-related foot disease (DFD), including foot ulcers, infection and gangrene, is one of the 10 leading causes of the global disability burden [1]. About 40% of people who develop DFD experience a recurrence within one year, and thus DFD represents a chronic disease; the focus of research into this should be on avoiding remission and preventing major consequences, such as amputation and death [2]. Key risk factors for DFD recurrence and complications in people at risk of DFD include high plantar pressures, abnormal gait, hyperglycaemia, hypertension and dyslipidemia [3,4,5]. Randomised controlled trials and meta-analyses show that foot disease is preventable by the control of these key reversible risk factors using interventions such as appropriate foot care, footwear and medical management [3,4,5,6]. A range of sensors and wearables have been developed or are currently under development for the remote monitoring of these key risk factors and this combined with telehealth management offers a way to remotely care for people at risk of DFD, as shown in Table 1. The implementation of these approaches could also minimize the risk to patients and staff of exposure to the current global SARS-CoV-2 pandemic [7,8].

**Figure 1:**
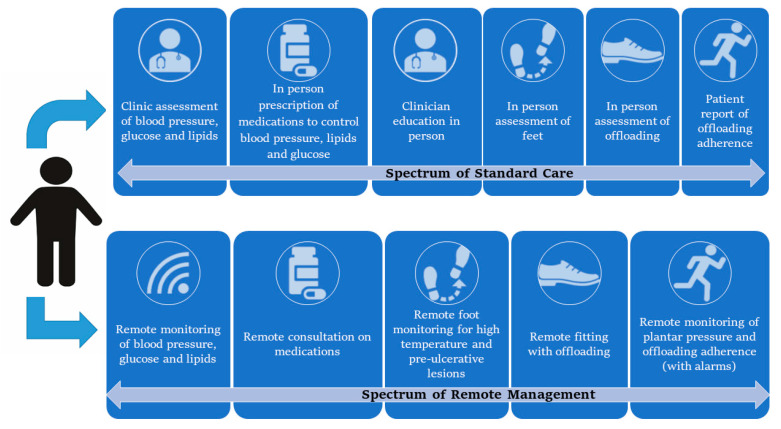
Key aspects of existing standard care compared with a future remote prevention program for diabetes-related foot disease. Legend: Comparison of existing in-person standard care of people with an at-risk foot (top care spectrum) compared with a future remote based management model (bottom care spectrum). The diagram outlines the key areas for prevention that can be targeted with sensors, wearables and telemedicine.

This review summarizes the potential application of remote monitoring systems using sensors and wearables to prevent DFD in the at-risk population, as shown in Figure 1 and Table 1. The challenges of implementing remote DFD prevention and how sensors and wearables could be applied to better prevent DFD are discussed below.

## 2. Monitoring Foot Temperature

Most foot ulcers develop due to repetitive trauma on the feet of people with a loss of protective sensation, such as those with diabetic peripheral neuropathy (DPN) [2]. Recurrent trauma results in local inflammation, or a “hot spot”, which can be detected by an elevated temperature at the affected site [16]. This offers a means to identify people who are likely to develop foot ulcers for immediate foot care, such as the removal of calluses and modifications of footwear, to achieve better offloading to reduce this repetitive trauma and in turn the hot spot [2]. Most previous studies have used infra-red thermometers to measure foot temperature at multiple sites on both feet and compare identical sites on opposite feet [17,18,19,20]. Prior research suggests that a temperature difference between identical sites on opposite feet of >2.2 °C (equivalent to ~4 °F) on two consecutive days can accurately predict ulcer development [21,22]. It has recently been reported that the difference between the median of temperature at six key locations on one foot (the hallux, first, third and fifth metatarsal heads, the mid-foot and heel) and ambient temperature is also able to predict foot ulcer development with an excellent sensitivity, although with limited specificity [23]. This potentially allows people with a unilateral foot ulcer and those with prior unilateral major amputation to also be monitored for the hot spots that are predictive of impending ulcers.

Given its predictive value, regular foot temperature monitoring offers the opportunity to instigate urgent offloading and foot care, such as callus removal, to prevent an impending foot ulcer. Four randomised controlled trials have examined the efficacy of daily home foot temperature monitoring to signal the need for offloading in people at risk of diabetes-related foot ulcers [17,18,19,20]. Three of these trials [17,18,19], which were performed by the same team and included a total of 427 participants, reported a significant and substantial reduction in foot ulcer incidence in those allocated to home foot temperature monitoring. The other trial [20], performed by a different research group and including only 41 participants, reported no significant effect of home foot temperature monitoring and urgent offloading on foot ulcer incidence [20]. A larger randomised trial involving 304 participants is currently examining the cost-effectiveness and cost-utility of home foot temperature monitoring [9]. Recently, a further clinical trial reported on the efficacy of foot temperature monitoring performed at only monthly intervals at an out-patient clinic, rather than at home [24]. A thermal camera was employed to identify “hot spots” in order to advise on interventions, such as reductions in physical activity and improved offloading of the concerned area [24]. The trial included 110 people with a past history of a diabetes-related foot ulcers and reported no benefit of the intervention in preventing foot ulcers or improving health-related quality of life [24]. It is possible that these contrasting findings relate to the less-frequent monitoring of foot temperature performed, which may have missed an opportunity for the early identification of at-risk patients. These findings suggest the potential benefit of applying modern technology to regularly monitor foot temperature remotely in the participant’s home in contrast to less frequent monitoring in outpatient clinics.

The International Working Group on the Diabetic Foot (IWGDF) recently gave only a weak recommendation for the use of home foot temperature monitoring based on the moderate quality of evidence [25]. This likely reflects the small size of prior trials, limitations in the design of the previous trials and the practical difficulties of implementing home foot temperature monitoring. The previous trials testing home foot temperature monitoring [17,18,19] have excluded people with peripheral artery disease (PAD), which is an established risk factor for foot ulceration, thereby limiting the generalizability [20,21,22]. Both PAD and DPN have been reported to influence foot temperature [23]. In a recent thermal imaging study, participants with PAD had a significantly higher foot temperature than those that did not have PAD [26,27]. In contrast, previous studies have reported a positive correlation between foot temperature and ankle brachial pressure index, implying that people with PAD have a lower foot temperature [28]. This disparity might relate to whether people with severe PAD are studied or not. Furthermore, prior clinical experience and recent reports of infrared thermography show that foot temperature rises immediately following successful revascularization in correlation with the increase in the ankle brachial pressure index [29]. Given the established effect of leg ischemia on foot temperature and the exclusion of participants with this problem from prior trials [17,18,19,20,24], the role of home foot temperature monitoring in people with PAD remains unclear. The ongoing trial of home temperature monitoring detailed above only excludes people with critical limb ischemia (defined as a systolic ankle blood pressure <50 mmHg, systolic toe blood pressure <30 mmHg or transcutaneous oxygen pressure <30 mm Hg) and therefore will better clarify the role of home temperature monitoring in people with milder forms of PAD [9]. 

Accepting the need for additional evidence for home foot temperature monitoring in larger numbers of people at risk of DFD with broader inclusion criteria, there are also some practical challenges to implementing this preventative approach. Previous trials have required participants to separately measure temperature at 12 locations on their feet using an infrared thermometer daily [9,17,18,19]. Importantly, all trials to date have used the same type of hand-held device, which has been found to be a time-consuming method and may not be feasible for the majority of people with diabetes, such as those that have impaired vision, impaired mobility or who have multiple comorbidities which all have impacts on self-care motivation. There is therefore interest in developing sensors to better automate home foot temperature monitoring to make this measurement much more user-friendly.

The most advanced, currently described system for the automated measurement of foot temperature is the Podimetrics Mat [23,30]. This is a wireless mat that is designed to remotely monitor the temperature of the plantar surface of the foot with minimal involvement from the patient [30]. If the mat is stepped on for a period of about 20 s, it automatically takes a thermogram of both feet. The thermogram accurately measures temperature over the range of 15 to 40 °C and transmits the data securely to an approved server managed by the manufacturer. Foot temperature asymmetry is automatically calculated based on the thermogram. In a prior study of 129 participants with a past history of diabetes-related foot ulcers, a temperature difference of 2.2 °C between common sites on both feet correctly predicted 97% of foot ulcers, with an average lead time of 37 days and a false-positive rate of 57% [30]. Increasing the temperature threshold to 3.2 °C decreased sensitivity to 70% but reduced the false-positive rate to 32%, with approximately the same lead time of 35 days. About 86% of the participants used the system at least 3 days a week. However, this device is not commercially available outside of the United States of America and may also be too expensive for individual use. 

Other options for remotely monitoring foot temperature include a thermal camera incorporated into a mobile phone, insole devices or optical fiber based smart textiles, such as smart socks or insoles [10,27,30,31,32]. Smart phone infrared thermal imaging cameras have excellent agreement with more established infrared imaging systems and thus appear suitable for use in clinical practice [33]. These devices may need more development to allow patients to use them easily at home, and the cost of such devices may be a potential limitation. Smart socks have been tested in small numbers of people with DPN and been shown to be able to accurately and repeatedly measure temperature at multiple sites on both feet [31,32]. However, whether it is feasible to use these regularly over a prolonged follow-up process is not currently clear and remains to be investigated. A large randomised trial of 300 participants with severe DPN is currently evaluating the effectiveness of daily home-based foot temperature measurements using an intelligent sensor-equipped insole combined with photo documentation in preventing foot ulcers [10]. Results from this trial will provide larger-scale evidence on the value of this approach.

## 3. Monitoring Plantar Pressures 

DPN leads to loss of intrinsic foot muscles and changes in foot shape [2]. These changes can promote areas of high pressure within the plantar surface of the feet during standing or walking [2]. A prior meta-analysis suggests that people with DPN and a history of foot ulcers have higher plantar pressures during walking than those with DPN who have not had an ulcer [32]. The IWGDF guideline strongly recommended that people with a history of foot ulcers use footwear designed to reduce their high plantar pressures [25]. Plantar pressures are traditionally measured in clinical practice using highly designed pressure plates or insoles with pressure sensors located within health care or research facilities [34]. Systems such as the Pedar^®^ (Novel, Munich, Germany) and F-Scan™ (Tekscan Inc, Boston, MA, USA) are now available that can reproducibly measure plantar pressures within footwear [35]. These systems have been used to confirm that therapeutic footwear is effective at reducing plantar pressures [35]. They are also being extended to measure plantar pressures and other tissue stress on the plantar surface of the feet during everyday activity [36]. Patient access to such systems is limited, however, as they are only available in a small number of research or clinical settings, require specific protocols for obtaining data and are not available for home monitoring [37]. These systems differ in relation to the types of sensors used to measure plantar pressure; for example, some have large capacitive sensors and others have smaller resistive sensors or piezoelectric sensors which are more temperature-sensitive. Therefore, the user should be familiar with the advantages and disadvantages of each system, as this determines the application and the quality of data obtained [36,38,39].

Smart insoles, such as the SurroSense Rx system (Orpyx Medical Technologies, Calgary, Alberta), have now been developed that can monitor plantar pressures and provide alerts directly to wearers [40]. This system consists of a pressure-sensing insole that contains eight pressure sensors: three positioned under the metatarsal heads, two under the lateral plantar surface, one under the heel, one under the hallux and one under the lessor toes. The wearer receives an alert when sustained pressure is detected (pressure exceeding 35–50 mmHg and lasting over a 15-min period) and a pressure map of each foot showing the area where pressure is sustained [40]. The alert thresholds are based on the integration of pressure data over time. The correct therapeutic response to the alert is the offloading of the area with sustained pressure within 20 min of detection. In a study of 17 people with a past history of foot ulcers, those receiving a great number of alerts (at least one alert every two hours) wore their offloading for longer and had better adherence in responding to alerts [40]. Most participants felt that the insoles were useful and achieved good performance [40]. 

In a recent clinical trial [41], 58 patients with DPN and a recent history of a plantar foot ulcers were studied. They were randomly assigned to either an intervention group that received audio-visual alerts via a smartwatch linked to the SurroSense Rx insole system and offloading instructions when aberrant pressures were detected or a control group that did not receive any alerts. This trial reported a 71% reduction in ulcer incidence in the intervention compared with the control group (incidence rate ratio 0.29, 95% CI, 0.09–0.93; *p* = 0.037) [41]. However, this trial had a small sample size and a large dropout of 35% during the wearing-in period of the insole system and a further 50% dropout in the intervention group during follow-up [42]. In addition, the pressure feedback system used recorded pressure at a low sample frequency and failed to measure peak pressures. Importantly, 89% of alerts were received during static weight bearing positions and only 11% during walking in the study [42]. Therefore, the usability and ease of implementing this type of device remains to be demonstrated in well-powered clinical trials. Further evidence is needed for the widespread adoption of home plantar pressure monitoring.

## 4. Offloading Footwear Adherence Monitoring

Therapeutic footwear specifically designed to the shape of the patients’ feet and targeted to reduce >25% of peak plantar pressures is an established part of the management of people with DPN [25]. Such footwear is strongly recommended by the IWGDF guideline [25]. However, prior randomised trials of offloading insoles or footwear have shown inconsistent results, with only four of the eight trials reporting a reduction in the incidence of foot ulcers [43,44,45,46,47,48,49,50]. A likely contributor to the inconsistent results of these trials is the variation in adherence to offloading. In one of the trials, for example, it was reported that custom-made offloading footwear did not significantly reduce foot ulcer incidence [49]; however, among the 71 participants that adhered to their custom-made offloading footwear for 80% of the time they were weight bearing, there was a significant reduction in the incidence of foot ulcers of 50% compared to the control group [49]. This emphasizes the importance of adherence to wearing offloading footwear in order for it to be effective.

In order to facilitate footwear use, accurate and objective data on adherence are needed. Temperature sensors (thermistors) placed inside therapeutic footwear have been used to monitor offloading use [49]. Combining such sensors along with activity monitors allows footwear adherence as a proportion of daily weight-bearing activity to be estimated [49]. A previous study showed that a temperature threshold of 25 °C to indicate that footwear was worn had a sensitivity of 95%, a specificity of 99%, a negative predictive value of 99% and a positive predictive value of 99% in determining footwear use [51]. Past research also demonstrates that these temperature sensors are a valid method of estimating footwear adherence compared to adherence measured using a time-lapse camera [52]. It is anticipated that small sensors will soon become available that can accurately monitor an individual’s footwear use and activity and provide “live” data seamlessly to remotely located health practitioners [53]. This objective footwear adherence data may be used to inform remotely delivered motivational interviewing aimed at increasing the frequency of offloading [54]. Further developments are needed before this approach can be implemented in a remote management program. It should be noted that there is a paucity of commercially available sensors for monitoring adherence easily and accurately, and most of the aforementioned sensors have only been used in a research context [36,37,38,39,40,41,42,43,44,45,46,47,48,49,50,51,52,53,54,55].

## 5. Remotely Monitoring Medical Management

The optimal control of glucose, blood pressure and lipids is frequently not well implemented among people that develop DFD [56]. People with DFD have an increased risk of all-cause mortality (relative risk (RR) 1.89, 95% confidence intervals (CI) 1.60, 2.23) and fatal myocardial infarction (RR 2.22, 95% CI 1.09, 4.53) compared to people with diabetes without DFD [57]. In people with a history of diabetes-related foot ulcers, the risk of cardiovascular mortality is about 50% over 10 years and the annual mortality rate is estimated to be about 6% [58]. This emphasizes the importance of optimizing medical management in this population. 

Glycaemic control is important for preventing both macro and microvascular complications, and a meta-analysis of past randomised trials suggests that intensive glycaemic control prevents amputations [59]. In clinical practice, diabetes management is usually informed by self-monitoring of blood glucose [60]. Wearable or implantable sensors are now available for the continuous monitoring of glucose [60]; these use enzymatic technology to monitor interstitial fluid rather than blood glucose [61]. These sensors can measure glucose up to every 5 min non-invasively for a period of about one week, after which most devices need to be replaced [56]. Such sensors have been incorporated into closed loop systems which provide automated insulin delivery to people with type 1 diabetes with improvements in glycaemic control [62]. Recent meta-analyses of randomised trials comparing self-monitoring and the continuous automated monitoring of glucose in people with type 2 diabetes suggest that continuous monitoring facilitates better glycaemic control [61,63,64,65]. The use of such devices is now recommended by the North American guidelines for some patients, such as those with poor glycaemic control (HbA1c ≥ 9%) [66]. A recent trial showed that flash glucose monitoring (measuring interstitial fluid glucose) can be implemented in the primary care environment, although it may not be superior to traditional methods as measured by HbA1c at 12 months [12]. The application of continuous glucose monitoring for people with diabetes who are at a high risk of complications such as DFD may have substantial benefits, but access to this technology is currently limited to selected patients due to the current high expense of such monitoring systems.

High blood pressure is another important risk factor for complications in people with DFD. Anti-hypertensive medications, such as angiotensin-converting enzyme inhibitors and angiotensin receptor blockers, have been shown to reduce the incidence of cardiovascular events in people at risk of DFD, such as those with PAD [67]. Control of blood pressure is, however, frequently suboptimal in people at risk of DFD [68]. In a recent study of 2773 people with PAD, about 40% had a systolic blood pressure above the target level of 140 mmHg [68]. Currently, blood pressure is monitored through the assessment of pulsation linked with an inflatable cuff placed around the upper arm. Novel cuff-less wearable devices have now been developed for the estimation of blood pressure and may provide a more practical way of repeatedly monitoring blood pressure and facilitating better management [13,69]. These devices use varying methods, such as pulse transit time, laser Doppler flowmetry and artery vibration, to calculate blood pressure. Some of these devices are available commercially, such as from TMART Technologies Limited, China and Somnomedics, Germany, and some—but not all—have been shown to accurately measure blood pressure in small numbers of people with comparable results to classical cuff-dependent machines and also intra-arterial assessments [69,70,71]. The accuracy and value of these devices in improving the medical management of people at risk of DFD need further evaluation.

People at risk of DFD also require lipid control. The intensive lowering of low-density lipoprotein has proven efficacy in reducing major adverse cardiovascular and limb events in people at risk of DFD, such as those with PAD and diabetes [4,72]. Low-density lipoprotein sensors have also been built, although further development and testing is needed before they will be ready for widespread use [73]. 

Medication non-adherence is often defined as taking less than 80% of the prescribed treatment [74]. Due to a variety of factors including cost and regimen complexity, adherence to diabetes treatment is often poor and is reported to vary from 23% to 77% across differing populations [75,76]. In order to achieve optimal control of risk factors, it is important that patients adhere to prescribed medications. Sensors have now been developed that are capable of monitoring medication ingestion; for example, Proteus Discover provides data on medication taking and physical activity to both patients and practitioners [77]. It consists of an ingestible sensor, a wearable sensor patch, a patient mobile app and a provider Web portal. After being swallowed, the ingestible sensor is activated and sends a signal with a specific code that is detected by the patch. When the ingestible sensor pill is taken with medication, it can measure medication ingestion adherence. The patch also can measure activity, heart rate and step count. Data from the patch are transmitted to a mobile device to be viewed by the patient and then to the Cloud and onto a Web portal for a practitioner to view. The mobile device app prompts the patient to take their medication doses as scheduled. A previous study suggested that Proteus Discover can improve control of blood pressure, low-density lipoprotein and HbA1c [77]. Such sensors could have a role in people at risk of DFD, but this needs further testing and consultation with patients and other key stakeholders. There is a lack of head-to-head clinical trials comparing the various types of sensors or monitors available for remote medical management described above; more importantly, the control arms in clinical trials of remote monitoring systems have varied substantially. Therefore, there is an ongoing need to assess the suitability of these sensors for optimizing medical management in people at risk of DFD.

## 6. Wearables for Assessing Sensation, Peripheral Perfusion and Gait 

People with DPN often have an abnormal gait, which likely contributes to high plantar pressures and the risk of foot ulcers [78]. The assessment of gait is complex, but wearables have now been developed that are capable of monitoring it remotely [79]. Such information can be potentially used for the design of a gait retraining program aimed at reducing plantar pressures and risk of ulceration. Artificial intelligence systems, such as the Gait-Enhancing Mechatronic System (GEMS) (Samsung, Seoul, Korea), have also been developed to improve gait and redistribute foot pressure, although their exact role in health care is not currently clear [80]. Other web-based and remotely delivered methods of physical therapy and rehabilitation may provide further ways to improve gait in people with DPN [81]. A clinical trial is currently in progress to test whether a remotely delivered physical therapy program can improve DPN symptoms and severity as well as gait and function [14]. 

Artificial intelligence has also been applied to the development of a robotic systems for assessing DPN that are potentially able to screen people for their risk of developing DFD [82]. There is a current Cochrane review that is reviewing the evidence of the accuracy of all potential simple tests for screening DPN to supply more comprehensive evidence [83]. There is also interest in the development of sensors to determine foot perfusion [15]; the vascular early warning system (VEWS), for example, functions by using infrared optical sensors placed on the toe and dorsum of the foot to measure changes in blood volume within the microvasculature during foot elevation [15]. These sensors have not been tested in well-powered randomissed clinical trials or in comparison to standard care [84]. One challenge with assessing these systems in clinical contexts is that standard care can vary substantially between health services, countries and continents; therefore, the role of such systems in delivering preventative medicine remains unanswered but is an exciting future area of research. It is possible that remotely delivered gait rehabilitation programs may reduce the risk of DFD.

## 7. Telehealth

For people with DFD, treatment and education typically occur in an outpatient clinic weekly or bi-weekly. Although remote monitoring methods for people with DFD using smartphone applications have been developed, these are still in their infancy and have not been widely tested or implemented [85,86,87]. Despite their potential application in remote DFD monitoring, the diagnostic accuracy of mobile phone images is reported to be poor and therefore should not be used as a stand-alone diagnostic instrument for DFD [88]. This is a rapidly evolving area; therefore, novel mobile phone applications and remote monitoring methods may improve over time. 

Telehealth is an established means of performing medical consultations [89]. The benefit of using telehealth for managing foot ulcers has been demonstrated in several meta-analyses and systematic reviews [90,91,92]. Most of the evidence comes from two clinical trials [93,94]: the first trial evaluated the effectiveness of a telehealth intervention made up of 2:1 online:standard outpatient consultations compared to a usual care intervention consisting of three standard outpatient clinic visits on ulcer healing in 374 people [93]. The authors reported no significant difference in ulcer healing or amputation between the two methods but did show an increased risk of mortality in the remote monitoring group (HR = 8.68, 95% CI: 6.9–10.88). This was a surprising finding that was not explained by any of the studied covariates [93].

A more recent cluster randomised controlled non-inferiority trial of 182 adults evaluated telehealth [94]. Weekly telemedicine consultations via an interactive Web-based ulcer record and a mobile phone for communication with the healthcare specialist in addition to outpatient clinic visits every 6 weeks was compared to visiting the outpatient clinic every second week [94]. The trial showed no difference in time to ulcer healing and a superiority in prevention against amputation (mean difference: 8.3%, 95% CI: 16.3%, −0.5%) in the intervention group [94]. An important factor to note in these trials was that the intervention arms all included some face-to-face consultations with a health care professional. Based on anecdotal evidence, at present, there appears to be a range of different approaches to telemedicine that are used globally, ranging from mobile phone-based consultations, hospital-based remote management consultations and the phone-based review of patients. However, how such approaches should be designed in line with face-to face care has not been well defined in the literature. 

There has been limited study of the value of telehealth consultations in preventing rather than treating DFD. The COVID-19 pandemic has provided a stimulus for studies testing the use of remote monitoring technologies and telehealth consultations in preventing DFD [7,8] (see Table 2). 

## 8. Conclusions

Sensors, wearables and telehealth approaches capable of remotely monitoring the key risk factors for DFD have been developed. We believe that the utilization of sensors, wearables and telemedicine approaches outlined in this review—and those currently under development—will offer an innovative means to approach the assessment of risk factors in people with DFD. It remains to be seen what broad impact these can have on the prevention of DFD. The COVID-19 pandemic may provide the stimulus for the innovative and pragmatic large-scale testing of a technological approach to preventing DFD in efforts to keep feet safe, intact and at home.

## Figures and Tables

**Table 1:** Examples of sensors and wearables with potential value for preventing DFD.

Risk Factor	Current Management Approach	Sensors or Wearable Devices	References	Potential Value of Sensor/Wearable	Potential Impact on Prevention
Pre-ulcerative lesions	Visits to podiatrist	Home foot temperature monitor and mobile phone applications	[9,10]	Offloading of “hot spots” following confirmed persistent temperature differences	Reduced progression of at-risk sites prone to develop foot ulcers
Elevated plantar pressures	Offloading footwear	Plantar pressure monitor	[11]	Warning systems to stimulate offloading and better design and modification of footwear	Improved offloading with reduced ulcer development
Elevated plantar pressures and tissue stress	Patient education	Footwear adherence monitor	[11]	Behaviour change support counselling informed by objective data	Improved offloading adherence
Hyperglycaemia	Capillary glucose monitoring	Continuous glucose monitor	[12]	Intensive glycaemic control	Better informed management of hyper and hypoglycaemia and reduced progression of macro and microvascular disease
Hypertension	Outpatient blood pressure measurement	Cuff-less blood pressure monitor	[13]	Better implementation of anti-hypertensive medications and more frequent monitoring	Better informed management of blood pressure and reduced progression of macro and microvascular disease and mortality
Abnormal gait	Not routinely managed	Gait and activity monitor	[14]	Gait retraining and encouraging remote physical activity	Reduce gait abnormalities potentially reducing plantar pressures and ulcer incidence
Peripheral artery disease	Vascular laboratory assessment using ultrasound or Doppler	Foot blood supply sensor	[15]	Earlier identification of complications and prompt medical management	Reduced progression of macro and microvascular disease

Legend: The table outlines the risk factors for the development of diabetes-related foot disease and how sensors and wearables could be used to remotely monitor these factors. References are provided for the relevant research articles assessing the impact or implementation of such technologies for further reading.

**Table 2:** Currently available and required evidence for the remote assessment and prevention of diabetes-related foot disease.

Remote Monitoring	Available Evidence	Current Limitations of Available Evidence	Relevant Studies
Home foot temperature monitor	A number of small RCTs show a decreased incidence of foot ulcers in people performing home-based temperature monitoring	Lack of a widely tested and user-friendly way of identifying “hot spots” Generalizability from prior smaller studies in select populations	[9,10]
Plantar pressure monitor	Possible to monitor plantar pressure remotely and use patient alarms to warn patients of impending sites of tissue breakdown as reported in one small RCT	Unclear if technology can be further developed to be more user-friendly and whether the findings are applicable and would be effective on a widespread basis	[11]
Footwear adherence monitor	Technology has been developed to accurately measure footwear adherence	Need for widespread testing of value of using devices Patients’ views on use of adherence monitoring is still unclear	[11]
Continuous glucose monitor	Highly developed area of monitoring and tested in multiple RCTs with proven benefit in improving glycaemic control (HbA1c)	Whether this remote monitoring improves outcomes in people at risk of developing DFD remains unclear	[12]
Cuff-less blood pressure monitor	Technology developed to assess this reported to be accurate in a small number of studies	Currently unclear whether these devices can be used on a widespread scale	[13]
Foot blood supply and sensation assessment	Technology still in the early developmental stages for monitoring	The benefit of these devices in improving clinical outcomes need to be further evaluated in RCTs	[15,82]

Legend: PAD = peripheral artery disease, RCT = randomised controlled trial, HbA1c = glycated haemoglobin A1c, DFD = diabetes-related foot disease.
